# Supplementary material for: Crawling and Gliding: A Computational Model for Shape-Driven Cell Migration
Source: PLoS Comput Biol. 2015 Oct 21;11(10):e1004280. doi: 10.1371/journal.pcbi.1004280 (PMC4619082; doi:10.1371/journal.pcbi.1004280)
Supplement: S1 Code — (ZIP) [file pcbi.1004280.s012.zip › release/tst/doc/html/classPDE.html]

Tissue Simulation Toolkit: PDE Class Reference


Tissue Simulation Toolkit
 0.1.4.1


- Main Page
- Namespaces
- Classes
- Files

- Class List
- Class Hierarchy
- Class Members

Public Member Functions |
Protected Member Functions |
Protected Attributes |
Friends |
List of all members

PDE Class Reference

`#include <pde.h>`

|  |  |
| --- | --- |
| Public Member Functions | |
|  | PDE (const int layers, const int sizex, const int sizey) |
|  | Constructor for PDE object containing arbitrary number of planes. More... |
|  | |
| virtual | ~PDE () |
|  | |
| void | Plot (Graphics \*g, const int layer=0) |
|  | Plots one layer of the PDE plane to a Graphics window. More... |
|  | |
| void | Plot (Graphics \*g, CellularPotts \*cpm, const int layer=0) |
|  | Plots one layer of the PDE to a Graphics window, but not over the cells. More... |
|  | |
| void | ContourPlot (Graphics \*g, int layer=0, int colour=1) |
|  | Plots the PDE field using contour lines. More... |
|  | |
| int | SizeX () const |
|  | Returns the horizontal size of the PDE planes. More... |
|  | |
| int | SizeY () const |
|  | Returns the vertical size of the PDE planes. More... |
|  | |
| int | Layers () const |
|  | Returns the number of PDE layers in the PDE object. More... |
|  | |
| double | Sigma (const int layer, const int x, const int y) const |
|  | Returns the value of grid point x,y of PDE plane "layer". More... |
|  | |
| void | setValue (const int layer, const int x, const int y, const double value) |
|  | Sets grid point x,y of PDE plane "layer" to value "value". More... |
|  | |
| void | addtoValue (const int layer, const int x, const int y, const double value) |
|  | Adds a number to a PDE grid point. More... |
|  | |
| double | Max (int l) |
|  | Gets the maximum value of PDE layer l. More... |
|  | |
| double | Min (int l) |
|  | Returns the minimum value of PDE layer l. More... |
|  | |
| void | Diffuse (int repeat) |
|  | Carry out $n$ diffusion steps for all PDE planes. More... |
|  | |
| void | NoFluxBoundaries (void) |
|  | Implementation of no-flux boundaries. More... |
|  | |
| void | AbsorbingBoundaries (void) |
|  | Implementation of absorbing boundaries. More... |
|  | |
| void | PeriodicBoundaries (void) |
|  | Implementation of periodic boundaries. More... |
|  | |
| void | Secrete (CellularPotts \*cpm) |
|  | Reaction and interaction of CPM plane with PDE planes. More... |
|  | |
| double | TheTime (void) const |
|  | Returns cumulative "simulated" time, i.e. number of time steps \* dt. More... |
|  | |
| double | GetChemAmount (const int layer=-1) |
|  | Returns summed amount of chemical in PDE plane "layer". More... |
|  | |
| void | GradC (int layer=0, int first\_grad\_layer=1) |
|  | |
| void | PlotVectorField (Graphics &g, int stride, int linelength, int first\_grad\_layer=1) |
|  | |

|  |  |
| --- | --- |
| Protected Member Functions | |
| virtual int | MapColour (double val) |
|  | Used in Plot. Takes a color and turns it into a grey value. More... |
|  | |
|  | PDE (void) |
|  | empty constructor (necessary for derivation) More... |
|  | |
| virtual double \*\*\* | AllocateSigma (const int layers, const int sx, const int sy) |
|  | Allocates a PDE plane (internal use). More... |
|  | |

|  |  |
| --- | --- |
| Protected Attributes | |
| double \*\*\* | sigma |
|  | |
| double \*\*\* | alt\_sigma |
|  | |
| int | sizex |
|  | |
| int | sizey |
|  | |
| int | layers |
|  | |

|  |  |
| --- | --- |
| Friends | |
| class | Info |
|  | |

## Constructor & Destructor Documentation

|  |  |  |  |
| --- | --- | --- | --- |
| PDE::PDE | ( | const int | *l*, |
|  |  | const int | *sx*, |
|  |  | const int | *sy* |
|  | ) |  |  |

Constructor for PDE object containing arbitrary number of planes.

Parameters
:   |  |  |
    | --- | --- |
    | layers | Number of PDE planes |
    | sizex | horizontal size of PDE planes |
    | sizey | vertical size of PDE planes |

PRIVATE

References AllocateSigma(), alt\_sigma, layers, sigma, sizex, and sizey.

|  |  |  |  |  |  |
| --- | --- | --- | --- | --- | --- |
| PDE::~PDE | ( | void |  | ) |  |

virtual

References alt\_sigma, and sigma.

|  |  |  |  |  |  |
| --- | --- | --- | --- | --- | --- |
| PDE::PDE | ( | void |  | ) |  |

protected

empty constructor (necessary for derivation)

References alt\_sigma, layers, sigma, sizex, and sizey.

## Member Function Documentation

|  |  |  |  |  |  |
| --- | --- | --- | --- | --- | --- |
| void PDE::AbsorbingBoundaries | ( | void |  | ) |  |

Implementation of absorbing boundaries.

Called internally (optionally) by Diffuse().

References layers, sigma, sizex, and sizey.

Referenced by Diffuse().

|  |  |  |  |
| --- | --- | --- | --- |
| void PDE::addtoValue | ( | const int | *layer*, |
|  |  | const int | *x*, |
|  |  | const int | *y*, |
|  |  | const double | *value* |
|  | ) |  |  |

inline

Adds a number to a PDE grid point.

Parameters
:   |  |  |
    | --- | --- |
    | layer | PDE plane. |
    | x,y | grid point |
    | value | value to add |

References sigma.

|  |  |  |  |
| --- | --- | --- | --- |
| double \*\*\* PDE::AllocateSigma | ( | const int | *layers*, |
|  |  | const int | *sx*, |
|  |  | const int | *sy* |
|  | ) |  |  |

protectedvirtual

Allocates a PDE plane (internal use).

For internal use, can be reimplemented in derived class to change method of memory allocation.

References layers, MemoryWarning(), sizex, and sizey.

Referenced by PDE().

|  |  |  |  |
| --- | --- | --- | --- |
| void PDE::ContourPlot | ( | Graphics \* | *g*, |
|  |  | int | *layer* = `0`, |
|  |  | int | *colour* = `1` |
|  | ) |  |  |

Plots the PDE field using contour lines.

Parameters
:   |  |  |
    | --- | --- |
    | g | Graphics window. |
    | layer | The PDE plane to be plotted. Default layer 0. |
    | colour | Color to use for the contour lines, as defined in the "default.ctb" color map file, which should be in the same directory as the executable. Default color 1 (black in the default color map). |

References conrec(), max, Max(), min, Min(), sigma, sizex, and sizey.

|  |  |  |  |  |  |
| --- | --- | --- | --- | --- | --- |
| void PDE::Diffuse | ( | int | *repeat* | ) |  |

Carry out $n$ diffusion steps for all PDE planes.

We use a forward Euler method here. Can be replaced for better algorithm.

Parameters
:   |  |  |
    | --- | --- |
    | repeat | Number of steps. |

Time step dt, space step dx, diffusion coefficient diff\_coeff and boundary conditions (bool periodic\_boundary) are set as global parameters in a parameter file using class Parameter.

References AbsorbingBoundaries(), alt\_sigma, Parameter::diff\_coeff, Parameter::dt, Parameter::dx, layers, Parameter::periodic\_boundaries, PeriodicBoundaries(), sigma, sizex, and sizey.

|  |  |  |  |  |  |
| --- | --- | --- | --- | --- | --- |
| double PDE::GetChemAmount | ( | const int | *layer* = `-1` | ) |  |

Returns summed amount of chemical in PDE plane "layer".

Parameters
:   |  |  |
    | --- | --- |
    | layer | The PDE plane of which to sum the chemicals. layer=-1 (default) returns the summed amount of chemical in all planes. |

References layers, sigma, sizex, and sizey.

|  |  |  |  |
| --- | --- | --- | --- |
| void PDE::GradC | ( | int | *layer* = `0`, |
|  |  | int | *first\_grad\_layer* = `1` |
|  | ) |  |  |

Calculates the first and second order gradients, i.e. gradx, grady, gradxx, gradxy and gradyy and puts them in the next three chemical fields. Not currently used and might need some redoing. Make sure you have allocated sufficient fields (this method generates five planes).

Parameters
:   |  |  |
    | --- | --- |
    | layer | PDE plane of which to calculate the gradients (default 0) |
    | first\_grad\_layer | first plane of five in which to write the results (default 1). |

References Parameter::n\_chem, sigma, sizex, and sizey.

|  |  |  |  |  |
| --- | --- | --- | --- | --- |
| int PDE::Layers | ( |  | ) | const |

inline

Returns the number of PDE layers in the PDE object.

References layers.

|  |  |  |  |  |  |
| --- | --- | --- | --- | --- | --- |
| virtual int PDE::MapColour | ( | double | *val* | ) |  |

protectedvirtual

Used in Plot. Takes a color and turns it into a grey value.

Parameters
:   |  |  |
    | --- | --- |
    | val | Value from PDE plane. |

Implement this function in you main simulation code. See e.g. vessel.cpp.

Referenced by Plot().

|  |  |  |  |  |  |
| --- | --- | --- | --- | --- | --- |
| double PDE::Max | ( | int | *l* | ) |  |

inline

Gets the maximum value of PDE layer l.

Parameters
:   |  |  |
    | --- | --- |
    | l | layer |

Returns
:   Maximum value in layer l.

References max, sigma, sizex, and sizey.

Referenced by ContourPlot().

|  |  |  |  |  |  |
| --- | --- | --- | --- | --- | --- |
| double PDE::Min | ( | int | *l* | ) |  |

inline

Returns the minimum value of PDE layer l.

Parameters
:   |  |  |
    | --- | --- |
    | l | layer |

Returns
:   Minimum value in layer l.

References min, sigma, sizex, and sizey.

Referenced by ContourPlot().

|  |  |  |  |  |  |
| --- | --- | --- | --- | --- | --- |
| void PDE::NoFluxBoundaries | ( | void |  | ) |  |

Implementation of no-flux boundaries.

Called internally (optionally) by Diffuse().

References layers, sigma, sizex, and sizey.

|  |  |  |  |  |  |
| --- | --- | --- | --- | --- | --- |
| void PDE::PeriodicBoundaries | ( | void |  | ) |  |

Implementation of periodic boundaries.

Called internally (optionally) by Diffuse().

References layers, sigma, sizex, and sizey.

Referenced by Diffuse().

|  |  |  |  |
| --- | --- | --- | --- |
| void PDE::Plot | ( | Graphics \* | *g*, |
|  |  | const int | *layer* = `0` |
|  | ) |  |  |

Plots one layer of the PDE plane to a Graphics window.

Parameters
:   |  |  |
    | --- | --- |
    | g | Graphics window. |
    | layer | The PDE plane to be plotted. Default layer 0. |

References MapColour(), Graphics::Point(), sigma, sizex, and sizey.

|  |  |  |  |
| --- | --- | --- | --- |
| void PDE::Plot | ( | Graphics \* | *g*, |
|  |  | CellularPotts \* | *cpm*, |
|  |  | const int | *layer* = `0` |
|  | ) |  |  |

Plots one layer of the PDE to a Graphics window, but not over the cells.

Parameters
:   |  |  |
    | --- | --- |
    | g | Graphics window. |
    | cpm | CellularPotts object containing the cells. |
    | layer | The PDE plane to be plotted. Default layer 0. |

References MapColour(), Graphics::Point(), CellularPotts::Sigma(), sigma, sizex, and sizey.

|  |  |  |  |
| --- | --- | --- | --- |
| void PDE::PlotVectorField | ( | Graphics & | *g*, |
|  |  | int | *stride*, |
|  |  | int | *linelength*, |
|  |  | int | *first\_grad\_layer* = `1` |
|  | ) |  |  |

Plots a field of the first order gradients, i.e. gradx and grady; assumes you have called GradC before. Not currently used and might need some redoing.

Parameters
:   |  |  |
    | --- | --- |
    | g | Graphics window |
    | stride | Number of grid points between vectors (drawn as lines, currently. |
    | linelength | Length of vector lines, in pixels. |
    | first\_grad\_layer | first plane of two which contain the calculated gradients (default 1). |

References Graphics::Line(), sigma, sizex, and sizey.

|  |  |  |  |  |  |
| --- | --- | --- | --- | --- | --- |
| void PDE::Secrete | ( | CellularPotts \* | *cpm* | ) |  |

Reaction and interaction of CPM plane with PDE planes.

Parameters
:   |  |  |
    | --- | --- |
    | cpm | CellularPotts plane the PDE plane interacts with |

You should implement this member function as part of your main simulation code. See for an example vessel.cpp.

|  |  |  |  |
| --- | --- | --- | --- |
| void PDE::setValue | ( | const int | *layer*, |
|  |  | const int | *x*, |
|  |  | const int | *y*, |
|  |  | const double | *value* |
|  | ) |  |  |

inline

Sets grid point x,y of PDE plane "layer" to value "value".

Parameters
:   |  |  |
    | --- | --- |
    | layer | PDE plane. |
    | x,y | grid point |
    | value | new contents |

References sigma.

|  |  |  |  |
| --- | --- | --- | --- |
| double PDE::Sigma | ( | const int | *layer*, |
|  |  | const int | *x*, |
|  |  | const int | *y* |
|  | ) |  | const |

inline

Returns the value of grid point x,y of PDE plane "layer".

Warning, no range checking done.

Parameters
:   |  |  |
    | --- | --- |
    | layer | the PDE plane to probe. |
    | x,y | grid point to probe. |

References sigma.

|  |  |  |  |  |  |
| --- | --- | --- | --- | --- | --- |
| int PDE::SizeX | ( | void |  | ) | const |

inline

Returns the horizontal size of the PDE planes.

References sizex.

|  |  |  |  |  |  |
| --- | --- | --- | --- | --- | --- |
| int PDE::SizeY | ( | void |  | ) | const |

inline

Returns the vertical size of the PDE planes.

References sizey.

|  |  |  |  |  |  |
| --- | --- | --- | --- | --- | --- |
| double PDE::TheTime | ( | void |  | ) | const |

inline

Returns cumulative "simulated" time, i.e. number of time steps \* dt.

## Friends And Related Function Documentation

friend class Info

friend

## Member Data Documentation

double\*\*\* PDE::alt\_sigma

protected

Referenced by Diffuse(), PDE(), and ~PDE().

int PDE::layers

protected

Referenced by AbsorbingBoundaries(), AllocateSigma(), Diffuse(), GetChemAmount(), Layers(), NoFluxBoundaries(), PDE(), and PeriodicBoundaries().

double\*\*\* PDE::sigma

protected

Referenced by AbsorbingBoundaries(), addtoValue(), ContourPlot(), Diffuse(), GetChemAmount(), GradC(), Max(), Min(), NoFluxBoundaries(), PDE(), PeriodicBoundaries(), Plot(), PlotVectorField(), setValue(), Sigma(), and ~PDE().

int PDE::sizex

protected

Referenced by AbsorbingBoundaries(), AllocateSigma(), ContourPlot(), Diffuse(), GetChemAmount(), GradC(), Max(), Min(), NoFluxBoundaries(), PDE(), PeriodicBoundaries(), Plot(), PlotVectorField(), and SizeX().

int PDE::sizey

protected

Referenced by AbsorbingBoundaries(), AllocateSigma(), ContourPlot(), Diffuse(), GetChemAmount(), GradC(), Max(), Min(), NoFluxBoundaries(), PDE(), PeriodicBoundaries(), Plot(), PlotVectorField(), and SizeY().

---

The documentation for this class was generated from the following files:

- pde.h
- pde.cpp


---

Generated on Thu Aug 14 2014 22:04:01 for Tissue Simulation Toolkit by  

 1.8.6
